# Discrimination Improvement of a Gas Sensors’ Array Using High-Frequency Quartz Crystal Microbalance Coated with Polymeric Films

**DOI:** 10.3390/s20236972

**Published:** 2020-12-06

**Authors:** Marcos Rodríguez-Torres, Víctor Altuzar, Claudia Mendoza-Barrera, Georgina Beltrán-Pérez, Juan Castillo-Mixcóatl, Severino Muñoz-Aguirre

**Affiliations:** Facultad de Ciencias Físico-Matemáticas, Benemérita Universidad Autónoma de Puebla, Av. San Claudio y Río Verde, Col. San Manuel, CU, C.P. 72570 Puebla, Mexico; marcos.rodriguezt@alumno.buap.mx (M.R.-T.); valtuzar@fcfm.buap.mx (V.A.); cmendoza@fcfm.buap.mx (C.M.-B.); gbeltran@fcfm.buap.mx (G.B.-P.); jcastill@fcfm.buap.mx (J.C.-M.)

**Keywords:** quartz crystal microbalance, gas sensor, polymeric sensing films, high frequency, high sensitivity, volatile organic compounds, discriminant analysis, partition coefficient

## Abstract

The discrimination improvement of an array of four highly sensitive 30 MHz gas quartz crystal microbalance (QCM) sensors was performed and compared to a similar system based on a 12-MHz QCM. The sensing polymeric films were ethyl cellulose (EC), poly-methyl methacrylate (PMMA), Apiezon L (ApL), and Apiezon T (ApT) and they were coated over the AT-cut QCM devices by the drop casting technique. All the sensors had almost the same film thickness (0.2 μm). The fabricated QCM sensor arrays were exposed to three different concentrations, corresponding to 5, 10, and 15 μL, of ethanol, ethyl acetate, and heptane vapors. The steady state sensor responses were measured in a static system at a temperature of 20 °C and relative humidity of 22%. Our results showed that the 30-MHz sensors have a higher sensitivity than 12-MHz ones (around 5.73 times), independently of the sensing film and measured sample. On the other hand, principal component analysis and discriminant analysis were performed using the raw data of the responses. An improvement of the classification percentage between 12 MHz and 30 MHz sensors was found. However, it was not sufficient, especially for low concentrations. Furthermore, using partition coefficient and discriminant analysis (DA), an improvement of 100% classification of the three samples was achieved for the case of the 30-MHz sensor array.

## 1. Introduction

Volatile Organic Compounds (VOCs) are one of the leading causes of indoor air pollution problems. These pollutants are emitted from different sources such as the volatilization of detergents, spray painting industry, inks, chemical agents in hospital disinfection, and even cooking. They can negatively affect human health, through several symptoms or diseases, including headaches, nervousness, or even cancer [1,2,3]. Moreover, VOCs play an essential role in food quality control [4,5], explosives detection [6,7], and disease diagnosis, among others [8,9]. Thus, there is a need to develop reliable, accurate, and cheap gas sensors capable of measuring low concentrations and discriminating between different VOCs.

A variety of sensing methods such as electrochemical resistors, optical sensors, field-effect transistors (FETs), surface acoustic wave (SAW) sensors, surface plasmon resonance (SPR), and quartz crystal microbalance (QCM) have been used for VOCs analysis [10,11,12,13]. The QCM technique has gained significant interest in the community because of high sensitivity, fast response, and low concentration detection of VOCs. Polymeric sensitive and selective films deposited onto the electrodes of QCM devices have been used as an adsorption surface of VOC vapors. The accumulated mass adlayer over the polymeric sensitive films due to different vapor molecules’ sorption provokes a shift of the crystal resonance frequency [14,15,16,17]. A variety of applications have been reported, such as odorant biosensors working on both gas and liquid phases [18,19].

Several factors influence the detection sensitivity of QCM gas sensors such as the kind of sensitive polymer, the film thickness, the functional groups affinities of the polymeric material to VOCs, environmental conditions (temperature T and relative humidity RH), and the fundamental resonance frequency of the QCM [20,21,22,23]. According to Alassi et al., even though QCM sensors present some drawbacks, they are an excellent choice due to their high sensitivity and simple implementation [15]. The resonant frequency is crucial since it determines the sensitivity to mass changes over the QCM surface, i.e., the frequency shift due to the interaction with VOC molecules being proportional to the square of such a resonance frequency according to the Sauerbrey equation [24]. Therefore, the use of QCM with higher resonant frequencies has been investigated. The most commonly used resonant frequencies range from 5 MHz to 20 MHz with mass sensitivities ranging from 17.7 to 1.1 ng cm^−2^ Hz^−1^ [25,26,27]. However, to improve the sensitivity, it is desirable to increase the QCM resonance frequency, since, with 30 MHz QCM, we can attain a sensitivity of 0.5 ng cm^−2^ Hz^−1^, which would allow us to measure lower concentrations with better resolution in the sensor responses [28]. Furthermore, 30-MHz QCM sensors are not frequently reported in the literature due to their oscillation instability [29,30,31]. In this work, we used 30 MHz QCM to fabricate an array of VOCs sensors, and we could stably measure their responses.

On the other hand, the interactions of the sensing films with VOC vapors generate specific response patterns. The data patterns can be analyzed by using statistical analysis tools such as artificial neural networks (ANN), cluster analysis (CA), analysis of variance (ANOVA), principal component analysis (PCA), and discriminant analysis (DA) [30,32,33,34,35].

In this work, a comparison of the responses for two QCM gas sensor arrays with four different polymeric sensing films is described. The arrays are composed of 12 MHz and 30 MHz fundamental resonance frequency QCM sensors, respectively, and they were used to measure and classify some selected VOCs with specific characteristics. The studied VOCs were ethanol, ethyl acetate, and heptane, which are important air pollutants [36], and they were selected in order to cover different functional groups (alcohols, esters, and alkanes), polarities (high, medium, and low), and different solubility parameters. Moreover, the selected polymeric sensing films were ethyl cellulose and polymethyl methacrylate because of their high affinity to alcohols and esters, and, on the other hand, Apiezon L and Apiezon T because of their high affinity to alkanes.

We showed that the response of the 30-MHz QCM sensors array (30QCM-SA) is around 5.73 times larger than that of the 12-MHz QCM sensors array (12QCM-SA). Using PCA and DA, we were able to determine the responses’ correlation of the four sensors considering the concentration of the target compounds at controlled temperature and relative humidity. Finally, in order to eliminate concentration, sensing film thickness, and resonances frequency effects, we used the partition coefficient for the recognition and classification of these VOCs. The results showed that the sensitivity and the classification percentage were improved by using the 30QCM-SA.

## 2. Theory

### QCM Sensors, 12 MHz vs. 30 MHz

The sensor sensitivity depends significantly on the QCM operation frequency. It can be verified by the Sauerbrey equation (Equation (1)), where the frequency change (Δf) is proportional to mass changes (Δm) onto the QCM electrode surface [24].

(1) $$\Delta f=\;-\frac{2f_{0}^{2}}{\sqrt{\rho_{q}\mu_{q}}}\frac{\Delta m}{A},$$

where $f_{0}$ is the fundamental resonance frequency of the crystal, $\rho_{q}$ is the density, $\mu_{q}$ is the shear modulus of the quartz material, and A is the electrode area.

The sensing film average thickness (Δd) can be estimated using Equation (2), where the sensing film polymer density is $\rho_{f}=\frac{\Delta m}{A\Delta d}$.

(2) $$\Delta d=\;-\frac{\sqrt{\rho_{q}\mu_{q}}}{2}\frac{\Delta f}{f_{0}^{2}\rho_{f}}.$$

Thus, a ratio between the 30-MHz QCM and 12-MHz QCM resonance frequencies can be established by considering the physical parameters $\rho_{q}$ and $\mu_{q}\;\mathrm{constant}$, and, assuming that Δd is the same for both sensors, we obtain Equation (3).

(3) $$\frac{\Delta f_{30}}{\Delta f_{12}}=\;\frac{f_{30}^{2}}{\;f_{12}^{2}}\;,$$

where $f_{30}$ and $f_{12}$ are the fundamental resonance frequencies of the 30-MHz and 12-MHz QCM, respectively. The ratio defined in Equation (3) has a theoretical value of 6.25.

The partition coefficient logarithm $\ln\left(K\right)$ is frequently used to analyze gas sorption characteristics of sensing films [37]. *K* is defined by Equation (4).

(4) $$K=\frac{C_{f}}{C_{g}}\;,$$

where $C_{f}$ is the gas concentration inside the sensing film and $C_{g}$ is that in the air, calculated from Equation (5).

(5) $$C_{g}=\frac{22.4T_{a}\rho_{s}V_{l}}{273M_{w}V_{c}}\times 10^{3}$$

where $C_{g}$ is in ppm, 22.4 L/mol is the volume of 1 mol of an ideal gas at standard conditions of pressure and temperature (1 atm and 273 K), $T_{a}$ is the measurement temperature, $\rho_{s}$ is the density of the VOC sample in g/mL, $V_{l}$ is the volume of the injected VOC sample in μL, $M_{w}$ is the molecular weight of the VOC sample, and $V_{c}$ is the volume of the measurement chamber. In our case, this volume was 1 L.

In the case of QCM sensors, $C_{f}$ can be calculated using the resonance frequency shift $\left(\Delta f_{g}\right)$ due to a VOC vapor sorption onto the sensing film, the resonance frequency shift $\left(\Delta f_{f}\right)$ owing to the sensing film, and the polymer density $\left(\rho_{f}\right)$, as shown in Equation (6).

(6) $$C_{f}=\frac{\Delta f_{g}}{\Delta f_{f}}\rho_{f}$$

Substituting Equation (6) in Equation (4), we obtain Equation (7) to determine K [38,39].

(7) $$K=\;\frac{\Delta f_{g}\rho_{f}}{{\Delta f}_{f}C_{g}}.$$

$C_{g}$ was determined by the measurement chamber dimensions and the injected amount of liquid VOC sample, and $\Delta f_{g}$ was experimentally measured as the QCM sensor response.

## 3. Experimental

### 3.1. Materials

The polymeric materials used as sensing films were ethyl cellulose (EC, CAS 9004-57-3), poly(methyl methacrylate) (PMMA, CAS 9011-14-7), Apiezon^®^ grease L (ApL, CAS 1267-02-3), and Apiezon^®^ grease T (ApT, CAS 9064-45-3). The VOC samples measured were ethanol (EtOH, CAS 64-17-5), ethyl acetate (EtOAc, CAS 141-78-6), and heptane (hp, CAS 142-82-5). EC, PMMA, ApT, hp, and EtOAc were purchased from Sigma-Aldrich, while ApL, EtOH, and chloroform were purchased from Supelco Analytical Products, J.T. Baker, and Meyer Corporation, respectively. All reagents were of an analytical grade and used without any purification.

### 3.2. QCM Sensor

The gas sensors were fabricated using HC49U encapsulation, AT-cut, silver electrode QCM with the fundamental resonance frequency of 12 MHz and 30 MHz. For 12 MHz, the crystal wafer diameter was 9.7 mm and the electrode radius was 2.4 mm, while, for 30 MHz, the wafer diameter was 6.5 mm with an electrode radius of 1.25 mm. These electrode dimensions do not present a large deviation from the Sauerbrey equation, according to Huang et al. [27,40]. The electrode radius for 12-MHz QCM is large and, for 30-MHz QCM. Even though the electrode radius is small, the deviation decreases because of the high frequency. The QCM electrodes were cleaned in an ultraviolet-ozone (UV/Ozone) chamber (Bioforce Nanoscience) for 1 min and used immediately. The polymer materials were dissolved in chloroform at 1 mg/mL concentration and stirred for 5 min. Then, the sensing films were deposited over the QCM electrodes by the drop-casting method. Furthermore, 1 µL of the sensitive polymer material solution was deposited on both QCM electrodes. To eliminate the sensing film thickness effect, we deposited all the films with the same thickness at around 0.2 µm. After solvent evaporation (10 min), the fabricated sensors were stored in a desiccator at least 24 h before the first response measurement. After that, they were kept inside the desiccator and taken out only for response measurement. The impedance curves were measured using a setup that consisted of an oscilloscope (DSOX4024A, Keysight Technologies, Santa Rosa, CA, USA) and a waveform generator (33,600A Series, Keysight Technologies, Santa Rosa, CA, USA) connected to a voltage divider composed of a 1 kΩ series resistor with the QCM. A frequency scanning of a sinusoidal signal (10 Vpp) was applied to the voltage divider through this setup. The output signal was measured at the QCM and used to calculate the impedance.

### 3.3. QCM Sensor Response Measurement

**Figure 1:**
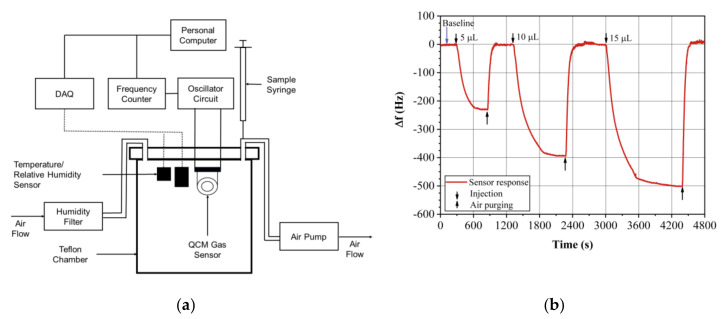
(**a**) Static set-up for measuring quartz crystal microbalance (QCM) gas sensors responses, (**b**) typical response curve of the QCM sensors, for this case a 30 MHz, AT-cut QCM coated with ethyl cellulose (EC) sensing film, and its response to EtOH.

The experimental setup used in this study is shown in Figure 1a. It is a static system to measure the steady-state response and consists of a measurement Teflon chamber connected to air inlet and outlet valves, respectively. It has an access port for the electrical connections of different devices such as gas sensors, humidity, and temperature sensors.

The QCM gas sensors were placed inside a Teflon chamber (1 L volume) together with a relative humidity sensor (HIH 4030, Honeywell, Charlotte, NC, USA) and a temperature sensor (LM-35, Texas Instruments, Dallas, TX, USA) for monitoring the environmental conditions. The QCM sensors were connected to an oscillator circuit located outside the chamber. The QCM frequencies were measured by a home-made frequency meter with a 1-Hz resolution and a sampling rate of 1 data per second [41]. The measured data of the sensor response, temperature, and humidity were stored on a personal computer using a type of software developed at our laboratory and reported in a previous work [42].

The VOCs’ liquid samples were injected into the chamber via a septum valve by employing a microliter syringe (50 µL, Hamilton, Reno, NV, USA) and waited until the steady-state has been reached. The purge of the chamber and the recovery process of the polymeric sensing films were carried out with an extraction pump (3.2 bpm, Micropump, HiLetGo, Shenzhen, China).

The QCM sensor responses were measured for three different concentrations corresponding to injections of 5, 10, and 15 μL of ethanol (2060, 4120, and 6180 ppm), ethyl acetate (1230, 2460, and 3690 ppm), and heptane (710, 1420, and 2130 ppm). The concentrations were calculated using Equation (5). All measurements were repeated five times at T=20 ± 0.5 °C and RH =22 ± 1%. A typical sensor response curve of EtOH for a sensor coated with EC is shown in Figure 1b. First, we waited for the baseline stabilization and, after that, we injected the liquid sample in the places marked with a downward arrow and we waited for the complete sample evaporation inside the chamber. After the response reached the steady state, it was read, stored in the computer, and a purge with dry air (marked with an upwards arrow) was performed to recover the baseline. This process was repeated for the three injections. The baseline was very stable and almost no drift was observed.

## 4. Results and Discussions

### 4.1. Atomic Force Microscopy (AFM) Images of the Sensing Films

**Figure 2:**
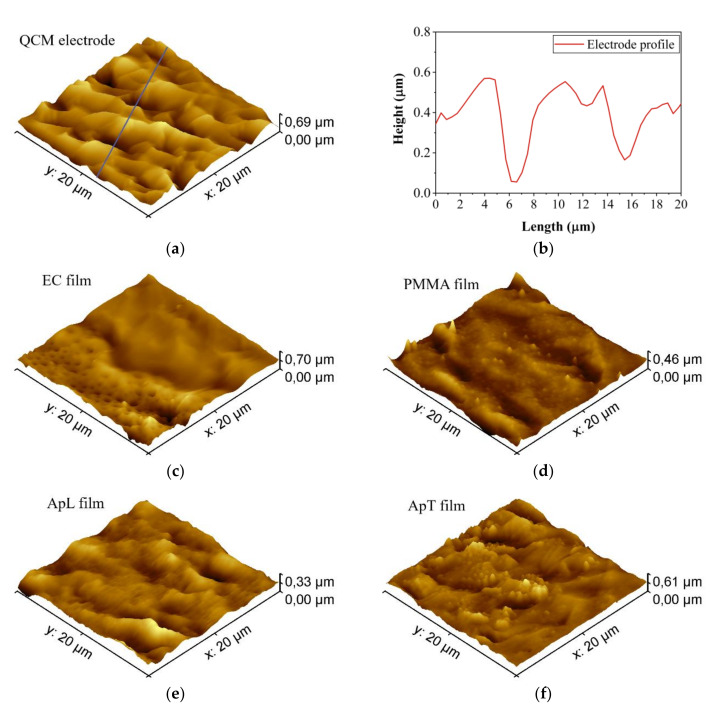
AFM images for (**a**) bare electrode, (**b**) bare electrode profile measured at the blue line of (**a**), (**c**) EC, (**d**) PMMA, (**e**) ApL, and (**f**) ApT sensing films deposited onto the QCM electrodes.

Typical tapping topography mode AFM images (AFM Park XE7, Park Systems Corp., Suwon, Korea), showing the bare QCM electrode, its profile as well as EC, PMMA, ApL, and ApT sensing films deposited on the QCM electrode, which are shown in Figure 2. The QCM electrode shows a mean roughness of 71.4 nm (Figure 2a,b). We observe the different morphologies after casting deposition of the sensing films (Figure 2c–f). In general, we can see that these films adopt the shape of the QCM electrode surface. Despite this, the morphology of the films differs from each other. Figure 2c shows the EC deposition on the electrode. A porous region is coated with the EC film (lower-left region) in contrast with the electrode without the polymer (upper-right region). On the other hand, PMMA, ApL, and ApT form a film covering the entire surface without pores. A porous film would allow us to have a larger contact area with the gases of volatile organic compounds. However, a further investigation is necessary to determine the influence of the sensing films’ morphology on the sensor responses, which is out of the scope of the present work.

### 4.2. Impedance Curves

**Figure 3:**
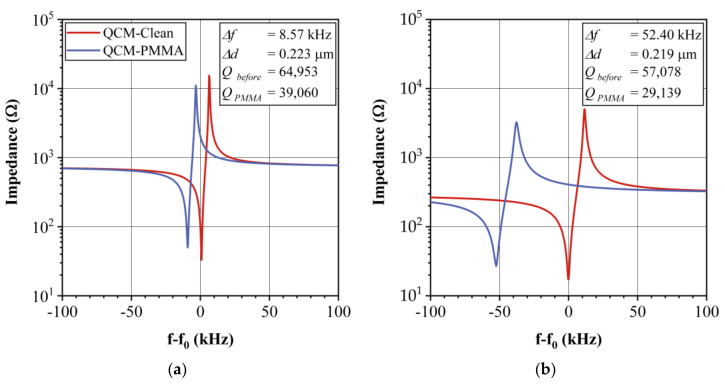
Impedance curves for a QCM without and with PMMA sensing films. (**a**) 12 MHz QCM and (**b**) 30 MHz QCM.

The electrical impedance curve of the coated QCM were measured to observe the frequency shift caused by the deposited polymeric sensing film. The measurement of impedance curves is necessary since they allow the observation of the stability and the effect of the sensing film deposition onto the QCM electrodes. Figure 3a,b show the impedance curves of the PMMA QCM sensor for both 12 and 30 MHz, before and after coating, respectively. The frequency shift (Δf) due to the sensing film deposition is more significant for the 30-MHz QCM sensor with a shift of 52.40 kHz, while that of the 12-MHz QCM sensor is only 8.57 kHz. The resonance peak sharpness and depth are related to the Q factor, which is related to the oscillation stability of the QCM since, when the peak is sharper, the oscillation circuit is better locked to the QCM frequency. The Q factor was calculated according to the method reported by Alassi et al. [15]. Generally, the 12-MHz crystals have a higher Q factor than that of 30-MHz crystals, and the effect of the sensing film deposition over this parameter is lower for the former, as can be observed in Figure 3. For 12 MHz, the Q factor, as calculated from the impedance curve electrical parameters, changes from 64,953 to 39,060 (ΔQ=40%), while, for 30 MHz, it changes from 57,078 to 29,139 (ΔQ=49%). The resonance resistance variation depends on the initial value before coating and, in general, the changes are lower for 12-MHz crystals. In Table 1, we show a summary of the Q factor and resonance resistance variations for the four sensing films used in this work. The 12-MHz sensors have a ΔQ from 4% to 40%, while for 30 MHz, such value is from 23% to 77%, depending on the kind of coating material. On the other hand, ΔR variations are less than 23 Ω for all the sensors, which indicates that their responses can be stably measured.

### 4.3. Comparison of Thickness and Sensitivity for 12-MHz vs. 30-MHz QCMs

Equations (2) and (3) were used to estimate the sensing film thickness (Δd) and calculate the ratio of the frequency shift ($\Delta f_{30}/\Delta f_{12}$) for each QCM with the same deposited polymeric sensing film. Such a ratio must be related to an increment of the gas responses. In Table 2, the sensing film parameters (density, thickness, frequency shift, and frequency shift ratios) are shown. The 30-MHz sensors’ thicknesses were normalized to those of 12-MHz ones, and then the Δf values were recalculated based on the linear behavior of QCM sensors. For instance, the Δf measured for EC (12 MHz) was 8100 Hz, which corresponds to a thickness of 0.218 μm, while, for the case of 30 MHz, the measured Δf was 39,600 Hz, corresponding to a film thickness of 0.171 μm. However, in order to perform a comparison, we must have the same thickness for both sensing films. Therefore, we recalculated Δf for 30 MHz assuming a thickness of 0.218 μm and we obtained the value 50,591 Hz, as is shown in Table 2. In all cases, the ratio between the frequency shifts is close to 6.25, the theoretical one, which is independent of the kind of material deposited on the QCM electrode, as is well known [43,44]. This result supports the fact that we can increase the QCM sensor sensitivity to different VOCs with an increment in the resonance frequency.

### 4.4. Sensitivity of 12-MHz vs. 30-MHz QCM Sensors

**Figure 4:**
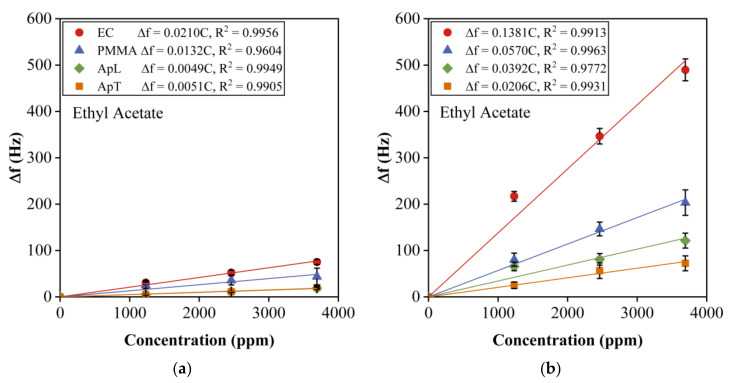
Response curves due to exposure to ethyl acetate (1230, 2460, and 3690 ppm) for sensors with EC, PMMA, ApL, and ApT sensing films, (**a**) 12 MHz, and (**b**) 30 MHz. The sensing film thickness was ~0.2 µm, at T=20 °C, and RH=22%. The maximum deviation was 13% error bars.

Figure 4 shows the average response (Δf) of the four sensing films (EC, PMMA, ApL, and ApT) due to the adsorption of ethyl acetate for the concentrations mentioned in Section 3.3. Figure 4a shows the results for 12 MHz, and Figure 4b for 30-MHz sensors. The response curves were measured five times for each sensor, and for each concentration value. The maximum deviation indicated by the error bars was 13%. In general, the response curves show a linear behavior, which is typical for these kinds of sensors, and the sensor sensitivities can be associated with the slopes of the calibration curves. We can observe that the sensor responses for 30-MHz sensors are higher than those of 12 MHz. For instance, EC has a response of 490 Hz for the maximum EtOAc concentration measured, while, for 12 MHz, such a value is 75 Hz. The fitting curves were calculated, and a linear correlation with $R^{2}$ between 0.9604 and 0.9963 was found, which indicates a very good fitting. On the other hand, sensitivity slopes from $S_{12}=0.0051$ Hz/ppm to $S_{12}=0.0210$ Hz/ppm and from $S_{30}=0.0206$ Hz/ppm to $S_{30}=0.1381$ Hz/ppm were found for 12-MHz and 30-MHz QCM sensors, respectively. Clearly, the sensitivities for 30-MHz QCM sensors is higher.

Moreover, the responses of ApL and ApT are very similar for 12-MHz sensors (19 Hz for ApL and 20 Hz for ApT). The fact that the responses are similar can cause EtOAc not to be classified correctly and that the array cannot distinguish between different VOCs. On the other hand, for 30-MHz sensors, the responses are different for those sensors (149 Hz for ApL and 72 Hz for ApT) and, since their values are higher, we can be confident that this better reflects the characteristics of the sensor array. EtOH and hp response curves behavior was the same for the four sensing films (data not shown).

**Figure 5:**
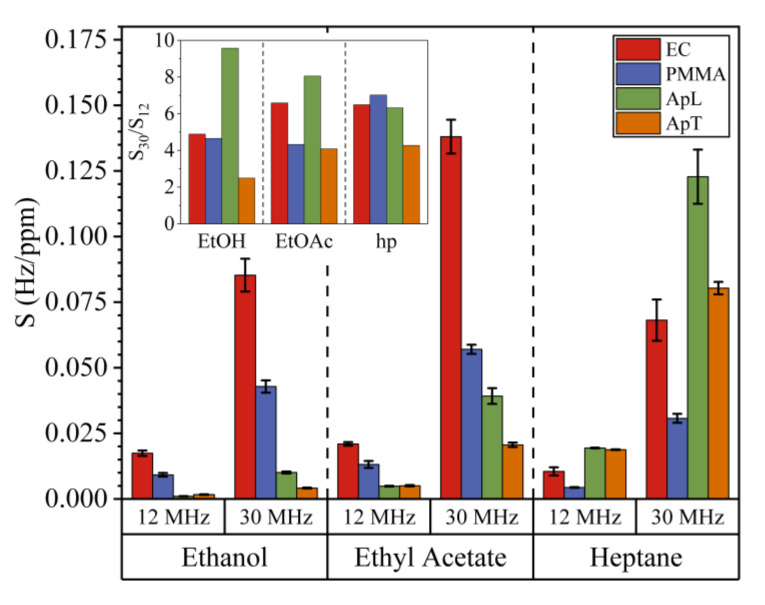
Comparison of sensitivities for 12QCM-SA and 30QCM-SA exposed to ethanol, ethyl acetate, and heptane. In the inset, the ratio between both sensitivities ($S_{30}/S_{12}$) is shown. The maximum deviation was 11% (error bars).

Figure 5 shows 12QCM-SA and 30QCM-SA average sensitivity values S (Hz/ppm) for EtOH, EtOAc, and hp for all sensing films. The maximum deviation indicated by the error bars was 11%. The inset shows the ratio ($S_{30}/S_{12}$) between sensors’ sensitivities, taking into account the target gas and the average for each sensing film. For EtOH values from 2.49 to 9.67, for EtOAc from 4.09 to 8.05, and hp values from 4.28 to 7.02. Theoretically, this value must be 6.25. However, the deviation is due to the different characteristics of the sensing films and the different response magnitudes. For instance, the sensitivity ratio for ApL exposed to EtOH is 9.67, which is much larger. We think this is due to the relatively low sensitivity of the 12-MHz sensor to this compound. In general, 30-MHz sensors have a higher average sensitivity value than 12 MHz, independently of the sensing film and compound.

The difference between all the sensitivity patterns is related to the sensing film affinity to the VOCs. The mutual solubility between the two materials is better since their solubility parameters δ are more similar. The sensing films’ solubility parameter are $\delta_{EC}=19.3\;{\mathrm{MPa}}^{1/2}$, $\delta_{PMMA}=20.87\;{\mathrm{MPa}}^{1/2}$, and $\delta_{ApL}=15.95\;{\mathrm{MPa}}^{1/2}$. We did not find in the literature any solubility parameter value for ApT. On the other hand, VOCs solubility parameters are $\delta_{EtOH}=26.52\;{\mathrm{MPa}}^{1/2}$, $\delta_{EtOAc}=18.15\;{\mathrm{MPa}}^{1/2}$, and $\delta_{hp}=15.30\;{\mathrm{MPa}}^{1/2}$. The EC and PMMA films have a higher response to EtOAc than hp, while, for ApL and ApT films, the opposite occurs. On the other hand, for EC and PMMA sensing films, their responses to EtOH are higher than those of ApL and ApT, while, for hp, the opposite is true. This behavior agrees with the matching between the solubility parameter values of sensing films with VOC samples. From these results, we could infer that the solubility parameter of ApT must be close to that of ApL [45,46,47].

### 4.5. Classification of VOCs by PCA and DA

**Figure 6:**
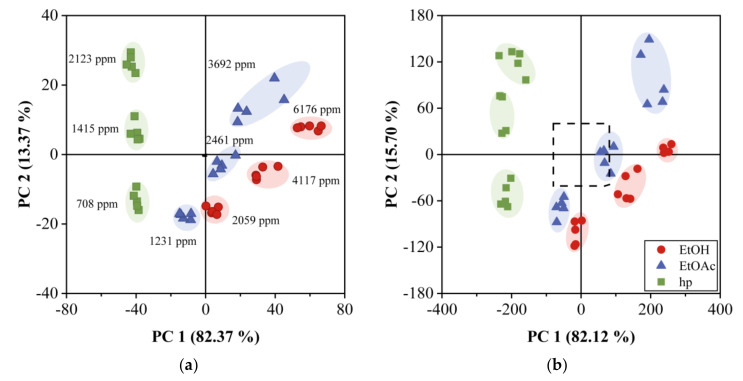
Principal component analysis for QCM gas sensor array response. (**a**) 12QCM-SA and (**b**) 30QCM-SA at T =20 °C and RH = 22%.

**Figure 7:**
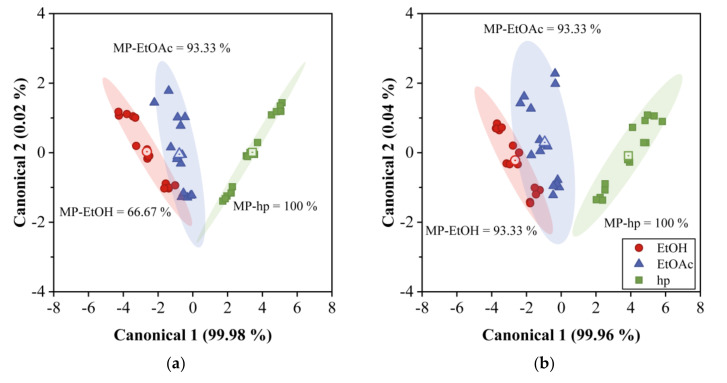
Discriminant analysis for QCM gas sensor array response. (**a**) 12QCM-SA and (**b**) 30QCM-SA at T = 20 °C and RH = 22%. Ethanol, ethyl acetate, and heptane data are enclosed by ellipses calculated at 2σ.

PCA and DA analysis were performed using the responses of both arrays to the three samples under study, and at the three concentration values mentioned above. The results are shown in Figure 6a,b, where the two first principal components scores are plotted for the 12QCM-SA and 30QCM-SA, respectively. The first principal component (PC1) accounted for 82.37% of the total data variance, while the second principal component (PC2) contained 13.37%. We observe that the three samples are distributed by the VOC type and concentration on the plot, hp on the left, EtOAc on the center, and EtOH on the lower right. The dotted region inside Figure 6b corresponds to the area of Figure 6a. We can clearly distinguish three groups for each sample for the three concentrations measured However, mainly for a low concentration range, EtOH and EtOAc regions overlap for the 12QCM-SA, while, for the case of 30QCM-SA, such regions are completely separated. This shows the effectiveness of the last array in performing the discrimination of the samples in the low concentration region due to the higher sensitivity. Such discrimination becomes evident when we perform DA, whose results are shown in Figure 7. Figure 7a shows the 12QCM-SA results, while those of 30QCM-SA are presented in Figure 7b. The data are enclosed by ellipses drawn at 2σ from each group center (dot-center symbol), which shows a 95% confidence. Here, EtOH is located on the left, EtOAc on the center, and hp on the right. Again, the low concentration regions overlap, owing to the lower sensitivity of the 12QCM-SA. DA gave us the membership percentage (MP) of the VOC to a sample group. From these results, the MP of EtOH was 66.67% for the 12QCM-SA, and 93.33% for 30QCM-SA. The remaining percentage can be confused with EtOAc for both arrays. In the case of EtOAc, MP was 93.33% for both arrays. Finally, in both cases, hp could be classified at 100%. In general, the MP values are higher for the 30QCM-SA, which means that it is more effective to discriminate the samples, especially for EtOH and EtOAc samples at low concentrations.

### 4.6. Partition Coefficient PCA and DA Results

**Figure 8:**
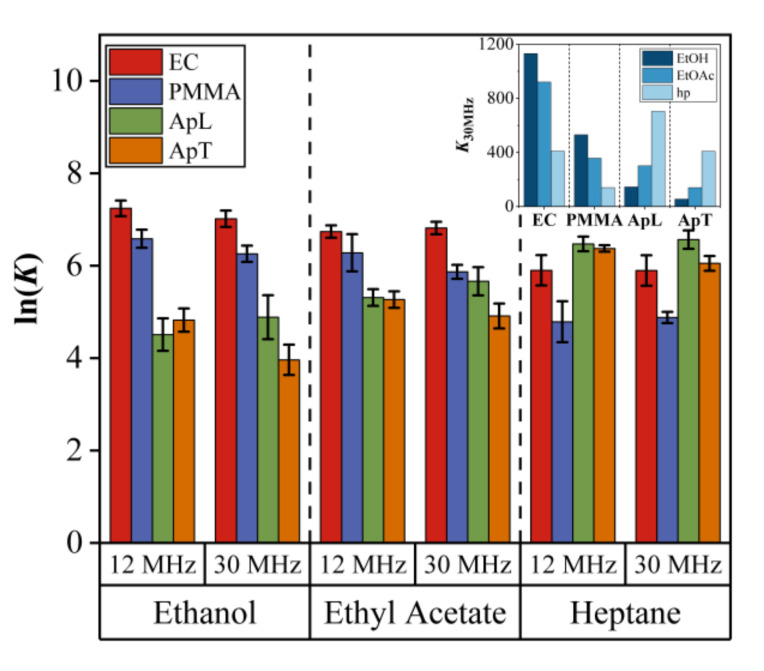
Comparison of the partition coefficient $\ln\left(K\right)$ for 12QCM-SA and 30QCM-SA. Maximum deviation of 10% (error bars).

The factors of concentration, sensing film thickness, and resonance frequency QCM can cause problems with the classification and recognition of the VOCs. Therefore, to eliminate such factors, a feature that can be used is the partition coefficient K, which is a descriptor of the sensing performance during absorption of a specific material with VOCs [14]. Moreover, this coefficient is dimensionless, as is shown in Equation (7), and, for high performance, it is usually associated with high sensor responses. The results of partition coefficient calculations, using lnK, are shown in Figure 8 for 12QCM-SA and 30QCM-SA. Since K is independent of concentration, film thickness, and QCM resonance frequency, the patterns are similar for both arrays with slight differences due to data dispersion and low responses of the 12QCM-SA (maximum deviation indicated by the error bars of 10%). For instance, for 12QCM-SA, the responses for ApL and ApT seems to be the same. However, for the pattern observed for 30QCM-SA, the responses for ApL are higher than those of ApT. Moreover, the responses are distributed according to the solubility parameter of the sample for both arrays. In the inset of Figure 8, we observe that EC and the PMMA sensor responses to EtOH are high, to EtOAc, are medium, and low to hp, while, for the case of ApL and ApT, the responses are in the opposite order. As it was mentioned above, this behavior is explained by the fact that the solubility parameters are very similar between EC, PMMA, EtOH, and EtOAc, which means high affinity of PMMA and EC to EtOH and EtOAc and low affinity to hp, while, for ApL and ApT, it is the opposite.

**Figure 9:**
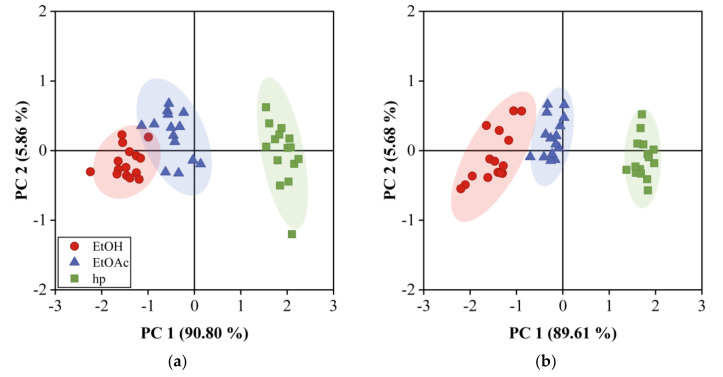
PCA results of partition coefficient $\ln\left(K\right)$ for QCM gas sensor array. (**a**) 12QCM-SA and (**b**) 30QCM-SA.

**Figure 10:**
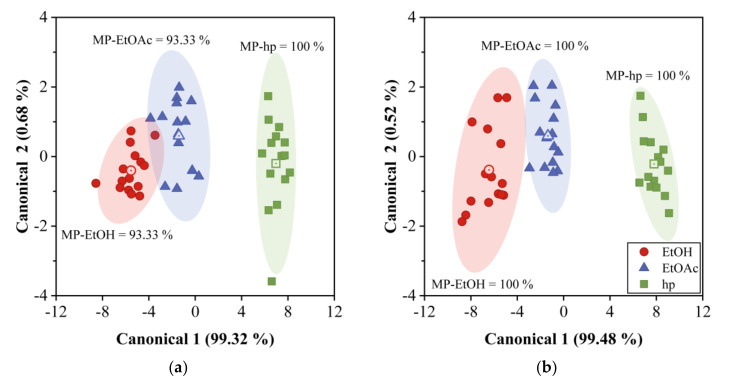
DA results of partition coefficient $\ln\left(K\right)$ for the QCM gas sensor array. (**a**) 12QCM-SA and (**b**) 30QCM-SA. Ethanol, ethyl acetate, and heptane data are enclosed by ellipses calculated at 2σ (95% confidence).

To assess the capability of QCM sensors’ array for discrimination between EtOH, EtOAc, and hp, PCA and DA were employed. Figure 9 shows the results for PCA using partition coefficient data. As can be observed, the points distribution is independent of concentration and we only have three data groups for the three VOC measured, EtOH, EtOAc, and hp. The groups of EtOH and EtOAc overlap (Figure 9a) for the 12QCM-SA, while they are completely separated in Figure 9b. Such a separation or discrimination can also be observed in Figure 10, where the results for DA are shown. In this case, the groups are enclosed by ellipses drawn at 2σ from each group center (dot-center symbol). If we compare Figure 10a with Figure 7a, we can see an MP increase from 66.7% to 93.33% for EtOH. On the other hand, in Figure 10b, the groups are completely separated and distributed in the plot according to the sample solubility parameter, EtOH is higher, and hp is smaller. Finally, for the 30QCM-SA, we have 100% MP for all the VOCs, which means the array successfully achieved the discrimination of the samples.

## 5. Conclusions

A study of the discrimination improvement of an array of high-frequency gas sensors based on 30-MHz QCM was performed using EC, PMMA, ApL, and ApT deposited over the QCM electrodes by the drop-casting method. The sensor responses were measured and they showed different sensitivities for ethanol, ethyl acetate, and heptane. Moreover, the obtained results were compared with a conventional 12-MHz QCM sensors array. A sensitivity increment was achieved using 30-MHz QCM devices. Theoretically, the increment must be 6.25. However, there are different aspects, such as sensing film structure, affinity, molecular weight, or environmental parameters (temperature and humidity) that change the sensitivity and selectivity behavior. We obtained an average sensitivity increment of 5.73.

PCA and DA results using raw data showed that, in general, MP values are higher for the 30QCM-SA, which means that it is more effective to discriminate the samples. Furthermore, the results using partition coefficient data revealed an evident increment in the array performance for 30 MHz QCM, obtaining a classification of 100% for the three measured samples.

## Figures and Tables

**Table 1:** Variations, before and after coating, of the Q factor and resistance for the QCM sensors.

Sensing Film	12 MHz				30 MHz			
	$Q_{before}$	ΔQ (%)	$R_{m,before}$ (Ω)	ΔR (Ω)	$Q_{before}$	ΔQ (%)	$R_{m,before}$ (Ω)	ΔR (Ω)
EC	92,369	13	22	2	59,033	23	15	7
PMMA	64,953	40	29	18	57,078	49	17	9
ApL	64,471	4	31	0.68	23,609	39	42	12
ApT	64,953	46	31	23	170,615	77	6	23

**Table 2:** Summary of the principal physical characteristics of each sensing film used in this work (density, estimated film thickness, frequency shift, and experimental frequency shifts ratio).

Sensing Film	ρ (g/cm^3^)	Δd (µm)	$\Delta f_{12}$ (Hz)	$\Delta f_{30}$ (Hz)	Experimental $\Delta f_{30}/\Delta f_{12}$
EC	1.14	0.218	8100	50,591	6.246
PMMA	1.18	0.223	8570	53,474	6.240
ApL	0.896	0.188	5490	34,296	6.247
ApT	0.912	0.200	5950	37,132	6.241
